# STAT3 signaling stimulates miR-21 expression in bovine cumulus cells during *in vitro* oocyte maturation

**DOI:** 10.1038/s41598-018-29874-w

**Published:** 2018-08-01

**Authors:** Allison Tscherner, Alyssa C. Brown, Leanne Stalker, Jennifer Kao, Isabelle Dufort, Marc-André Sirard, Jonathan LaMarre

**Affiliations:** 1 Department of Biomedical Sciences, University of Guelph, Guelph, ON Canada; 2 Centre de recherche en reproduction, développement et santé intergénérationnelle, Faculté des sciences de l’agriculture et de l’alimentation, Département des sciences animales, Pavillon des services, Université Laval, Québec, QC Canada

## Abstract

MicroRNAs are potent regulators of gene expression that have been widely implicated in reproduction and embryo development. Recent studies have demonstrated that miR-21, a microRNA extensively studied in the context of disease, is important in multiple facets of reproductive biology including folliculogenesis, ovulation, oocyte maturation and early mammalian development. Surprisingly, little is known about the mechanisms that regulate miR-21 and no studies have characterized these regulatory pathways in cumulus-oocyte complexes (COCs). We therefore investigated miR-21 in an *in vitro* model of bovine oocyte maturation. Levels of the primary transcript of miR-21 (*pri-miR-21)* and mature miR-21 increased markedly in COCs over the maturation period. Cloning of the bovine *pri-miR-21* gene and promoter by 5′3′RACE (rapid amplification of cDNA ends) revealed a highly conserved region immediately upstream of the transcription start site and two alternatively-spliced variants of *pri-miR-21*. The promoter region contained several putative transcription factor binding sites, including two for signal transducer and activator of transcription 3 (STAT3). Mutation of these sites significantly decreased both the intrinsic activity of *pri-miR-21* promoter-luciferase constructs and the response to leukemia inhibitory factor (LIF) (a STAT3 activator) in cultured MCF7 cells. In COCs, treatment with a STAT3 pathway inhibitor markedly decreased *pri-miR-21* expression and prevented cumulus expansion. *Pri-miR-21* expression was also inhibited by the protein synthesis inhibitor cycloheximide, suggesting that a protein ligand or signaling cofactor synthesized during maturation is necessary for transcription. Together these studies represent the first investigation of signaling pathways that directly influence miR-21 expression in bovine oocytes and cumulus cells.

## Introduction

Oocyte maturation consists of nuclear and cytoplasmic events that are required for the oocyte to acquire competency for fertilization, and subsequent embryo development should fertilization occur. Cumulus oophorus (typically known as cumulus) cells are an essential group of somatic cells surrounding the oocyte. They develop from granulosa cells into this specialized subtype during the antral stage of folliculogenesis^1–3^ (see review by^4^) and support the oocyte during maturation and fertilization. Removal of oocytes from their surrounding cumulus cells negatively impacts nuclear and cytoplasmic maturation and subsequent development to the blastocyst stage once the oocyte is fertilized^5–9^. The relationship between the oocyte and cumulus cells is multi-factorial and complex. During the final stages of maturation, cumulus cells propagate endocrine and biochemical signals to promote meiotic progression and the acquisition of oocyte competence while simultaneously responding to regulatory cues from the oocyte^10–12^ (reviewed by^13^). Many factors present in this microenvironment strongly influence cumulus cell gene expression, which in turn leads to the functional changes necessary for extracellular matrix synthesis, cumulus expansion, and the readiness of the cumulus oocyte complex (COC) for fertilization^14–16^.

Changes in gene expression that occur in cumulus cells during maturation include substantial modulation in the levels of microRNAs (miRNAs), an important class of endogenous small non-coding RNAs^17–19^ that have emerged as potent regulators of gene expression^20,21^ (see commentary by^22^). miRNA genes are highly conserved among eukaryotes and participate broadly in development, physiology, and pathology by influencing specific processes such as cell proliferation, differentiation, signal transduction and apoptosis^23^. miRNAs typically act by repressing the activity of target genes through anti-sense base-pairing with complementary sequences on the 3′ untranslated region (UTR) of messenger RNAs (mRNAs), causing translational repression or deadenylation and decay of these mRNA targets^24,25^ and resulting in post-transcriptional gene silencing. Comparative approaches, small RNA-sequencing and bioinformatics analysis have revealed that a substantial number of miRNA genes are expressed in bovine oocytes and their surrounding cumulus cells, a subset of which undergo dynamic changes in abundance over the course of *in vitro* maturation (IVM) and early embryonic development^26–29^. These profiles of global miRNA changes occurring in COCs have been useful for generating hypotheses regarding the potential regulatory function of miRNA during IVM, however mechanistic studies that establish particular roles for specific miRNAs are limited.

One miRNA that has been the subject of considerable research in the reproductive system and other contexts is miR-21, which plays an important, though incompletely understood role in the ovary. mir-21 is dynamically expressed throughout the estrus cycle^30^, and is the most abundant miRNA in bovine^29^ and ovine^30^ cumulus cells and among the three most abundant miRNAs in human cumulus cells^31^. miR-21 expression increases in ovine follicles throughout folliculogenesis, with a marked expression in growing follicles and a further increase observed in fully grown preovulatory follicles^30^. miR-21 is also abundantly expressed in periovulatory follicles in mice and furthermore, injection of miR-21 antagonists into murine ovarian bursa leads to increased apoptosis in cumulus cells and a reduction in number of recovered COCs in its corresponding oviduct, with a 50% decrease in ovulation suggesting important functional roles in ovulation and the regulation of apoptosis^32^. *In vitro*, a marked rise in miR-21 expression has been reported in cumulus cell and oocyte compartments during maturation in porcine COCs^33^, as well as in bovine oocytes and *in vitro* produced embryos, where miR-21 peaks at the 8-cell stage and is suspected to play a role in the degradation of maternal transcripts at the maternal zygotic transition (MZT)^26,34^.

Despite the obvious temporal associations with critical periods of oocyte and embryo development, the mechanisms directly controlling the induction of this gene in COCs are presently not well understood. miRNAs are first transcribed as long primary (pri-) transcripts that undergo sequential enzymatic cleavage steps before they are functional^35^. The initiation of miRNA processing is executed in the nucleus by the RNase III enzyme Drosha^36^, in conjunction with the RNA binding protein DiGeorge syndrome critical region 8 (DGCR8)^37,38^ that together form the minimal constituents of the “Microprocessor complex”^38,39^. In the present study we have examined *pri-miR-21* and miR-21 expression levels in order to identify factors and mechanisms that may participate in miR-21 induction and processing during IVM. We identify STAT3 as a potent activator of *pri-miR-21* transcription in cumulus cells. STAT3 acts as both a signal transducer and activator of transcription, and becomes activated as a DNA binding protein through tyrosine phosphorylation^40^. In response to cytokine signaling, STAT3 is typically phosphorylated and activated by a cascade that begins with ligand-initiated dimerization of receptor components, including the common signal transducer gp130^41^. These signals are propagated by the phosphorylation of Janus kinases (JAKs)^42^, which, in turn, activate STAT3 by phosphorylation. STAT3 is a major transcriptional activator downstream of cytokines from the gp130/interleukin-6 (IL-6) family, including IL-6^40^ and leukemia inhibitory factor (LIF)^43^, both of which are expressed in cumulus cells and are capable of inducing cumulus expansion^44,45^. We also document that miR-21 is induced under multiple IVM culture conditions after oocyte aspiration from follicles, and suggest that miR-21 is regulated by ligands synthesized and secreted by the COC itself.

## Results

### Primary *(pri)-miR-21* and mature miR-21 increase in bovine cumulus cells and oocytes during *in vitro* maturation of COCs

**Figure 1:**
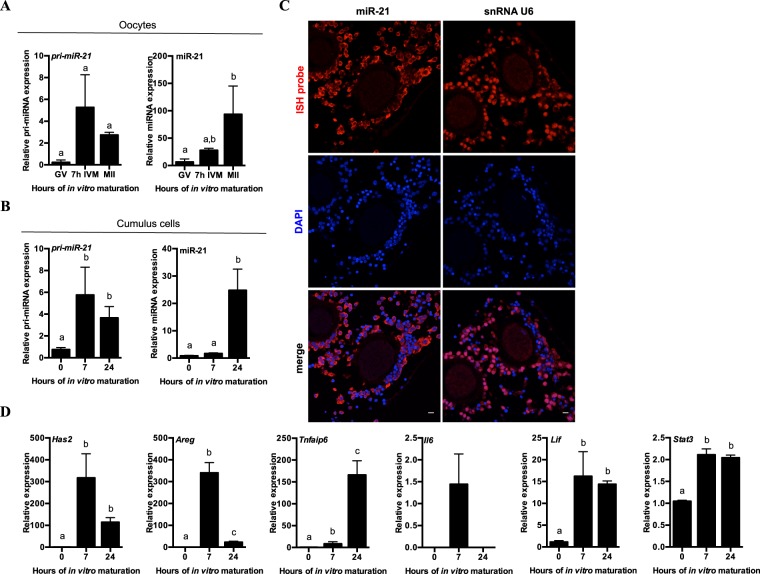
Primary miR-21 transcript and miR-21 are induced in bovine COCs during maturation *in vitro*. qRT-PCR quantification of *pri-miR-21* transcripts and mature miR-21 immediately after aspiration (0 hour) and at 7 or 24 hours of maturation in (**A**) oocyte (germinal vesicle (GV) to metaphase II (MII) stage) and (**B**) cumulus cell compartments of COCs. Gene expression is shown relative to the 0 hour or GV time point. Error bars represent SEM. N = 3 biological replicates. Different letters indicate statistically significant differences between groups. (**C**) RNA *in situ* hybridization of miR-21 or snRNA U6 in sectioned mature COCs. Nuclei counterstained with DAPI. Linear look-up tables. Scale bar represents 10 μm. (**D**) qRT-PCR quantification of cumulus expansion related transcripts and cytokine factors after aspiration (0 hour) and at 7 or 24 hours of maturation. Gene expression is shown relative to the 0 hour time point. Error bars represent SEM. N = 3 biological replicates. Different letters indicate statistically significant differences between groups.

To determine whether miR-21 and its precursor transcript, *pri-miR-21*, undergo dynamic changes in expression in bovine COCs throughout *in vitro* oocyte maturation, we first performed qRT-PCR on RNA isolated from pools of oocytes and cumulus cells collected at specific time points throughout that period. We have previously analysed miR-21 over the course of *in vitro* oocyte maturation (IVM) and fertilization^34^, and found a significant rise in oocyte miR-21 levels throughout IVM. We confirmed that finding here (Fig. 1A), in order to compare the oocyte expression profile with the expression of *pri-miR-21* and miR-21 in cumulus cells, which are expected to be a major contributor of miR-21 (Fig. 1B). *Pri-miR-21* transcript levels rose significantly in cumulus cells over the course of maturation, with an average increase of approximately 6-fold over the first 7 hours of maturation, followed by a slight decrease at 24 hours of maturation (Fig. 1B). The rise in *pri-miR-21* transcripts coincides with an overall 25–fold increase in mature miR-21 molecules from the beginning to end of IVM (Fig. 1B). In oocytes, an observable though not statistically significant change in *pri-miR-21* transcripts is seen between the germinal vesicle (GV) to metaphase II (MII) stage, yet a major change in mature miR-21 levels (~80-fold) was detectable at the end of the maturation period (Fig. 1A). In order to confirm this presence of mature miR-21 in the somatic and oocyte compartments of the COC after *in vitro* maturation, RNA *in situ* hybridization was performed with Locked Nucleic Acid (LNA) oligonucleotide probes specific for miR-21 or the small nuclear RNA (snRNA) U6, which was used as control (Fig. 1C). miR-21 and snRNA U6 are both detectable in the mature oocyte and its surrounding cells, in confirmation of the qRT-PCR results. miR-21 is strongly expressed in the cumulus compartment where it localizes to the cytoplasm as expected for a mature miRNA, while snRNA U6 is detected predominantly in cumulus cell nuclei. As an additional control, hybridization was performed with a scrambled miRNA probe (Supplemental Fig. 1). The scrambled probe did not have a complete absence of fluorescent signal, suggesting that its sequence might bind partially to some RNA sequences in the COC.

The decrease observed by qRT-PCR in cumulus cell *pri-miR-21* transcripts coincides with the observed marked rise in mature miR-21 at 24 hours (Fig. 1B), which is expected when transcripts are being actively processed by the Microprocessor. Similarly to cumulus cells, significant changes in mature miR-21 are not observed in oocytes until *pri-miR-21* becomes detectable. Peak *pri-miR-21* expression in cumulus cells occurs concurrently with an anticipated rise in the cumulus expansion gene *hyaluronan synthase 2 (Has2)*, the EGF factor *amphiregulin (Areg)*, and the cumulus expansion related gene *TNF alpha induced protein 6 (Tnfaip6)*, which undergoes further induction after the 7 hour time point (Fig. 1D). The interleukin 6 (IL-6) family cytokines *Il6* and *Lif*, as well as the downstream mediator of cytokine action *Stat3* also rise over this time.

### Full length primary miR-21 cloning identifies two distinct transcript variants that result from alternative splicing

**Figure 2:**
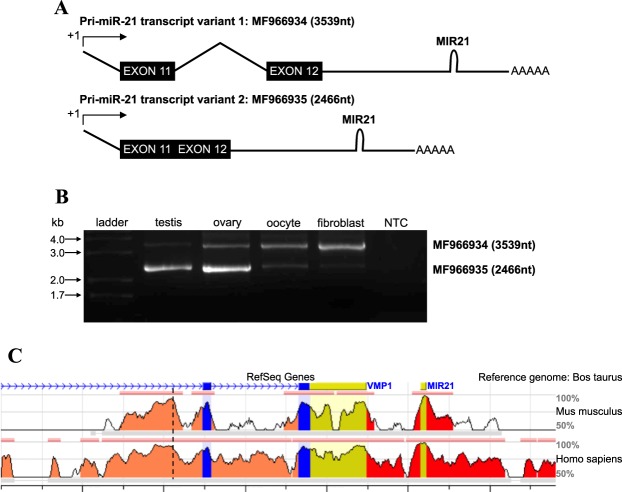
Bovine primary miR-21 exists as two transcript variants and contains a conserved putative upstream promoter region. (**A**) Predicted *pri*-*miR-21* transcript variants based on RACE-PCR derived sequences. (**B**) DNA gel electrophoresis of RT-PCR amplified *pri-miR-21* transcript variants in bovine tissues and cells. A very faint band representing *pri-miR-21* transcript variant 1 in testis is detectable. NTC; no template control. (**C**) *Bos taurus pri-miR-21* variant 1 plus 1 kb upstream region; alignment to *Mus musculus* Ch11 and *Homo sapiens* Ch17. Major ticks on x-axis = 1000 nucleotides, minor ticks = 500 nucleotides. Blue = coding exon, salmon = intronic region, yellow = untranslated region, red = intergenic region. Height of conservation plot at each position represents the number of nucleotides conserved out of a 100 nucleotide window centered at that position. Horizontal pink bars denote evolutionarily conserved regions (ECR) to the bovine reference genome. ECR defined as a minimum 70% identity over 100 nucleotide regions. Vertical hatched line denotes *pri-miR-21* transcription start site.

To begin to elucidate whether the observed changes in pri- and mature miR-21 expression in cumulus cells might be due to changes in precursor transcript expression, we first cloned and characterized the full-length primary miR-21 transcript in *Bos taurus* using a rapid amplification of cDNA ends (RACE) technique. Oligonucleotides designed to prime amplification of known segments of *pri-miR-21* (gene specific primers) were paired with oligonucleotides designed to prime RNA adapters ligated to the 5′ or 3′ *pri-miR-21* transcript ends. RT-PCR was then used to amplify this transcript from cDNA generated from bovine testis tissue, a rich source of *pri-miR-21* transcripts. Two 5′ end products and one 3′ end product were amplified, isolated and cloned into sequencing vectors. Sequence analysis of the bovine reference genome UMD 3.1.1 demonstrated that both of the amplified 5′ end products and the 3′ end product align exclusively to chromosome 19, beginning at position 11,030,051 and ending at position 11,033,585, where they flank the previously annotated *MIR21* gene (a 72 nucleotide hairpin containing mature miR-21) on the forward strand. The 5′ end products reside within an intron of the protein-coding gene vacuole membrane protein 1 (*VMP1*) between exons 10 and 11 of that gene, and the longer of the two products contains *VMP1* exon 11, intron 11–12, and exon 12. The shorter product aligns to the longer product with a 1072 nucleotide gap that represents the exclusion of *VMP1* intron 11–12, and the sequence reads directly from *VMP1* exon 11 to exon 12. A hexameric poly(A) signal (AAUAAA) was predicted on the 3′ end product by PolyA Signal Miner tool, at the expected location, 15 nucleotides upstream of the beginning of the sequenced poly(A) tail^46^. Together, these data were used to predict a 3538 nucleotide transcript and a 2466 nucleotide variant of the *pri-miR-21* transcript that would result from splicing (Fig. 2A). To confirm this prediction, cloning primers were designed near the 5′ and 3′ ends of the sequenced RACE products, and these primers were used to amplify full-length *pri-miR-21* from cDNA of bovine tissues and cells by RT-PCR. Both of the predicted variants are expressed in the testis, ovary, oocytes, and fetal fibroblasts (Fig. 2B). Since these transcripts can only be cloned from a cDNA template, the cloning products are slightly shorter than the actual transcripts (3458 and 2386 nucleotides, respectively) because suitable primers could not be placed at the extreme 5′ and 3′ termini. Sequencing analysis and alignment of cloned *pri-miR-21* variants with the bovine reference genome UMD 3.1.1 revealed two single nucleotide differences within the region corresponding to *VMP1* intron 11–12: two additional thymines are present at position 840–842, and a deletion of one thymine at position 1052 is evident, which together result in a transcript length 1 nucleotide longer than what we originally predicted based on the reference genome. The full sequence length of *pri-miR-21* is 3539 nucleotides, and not 3538 nucleotides as predicted. Both the 3539 nucleotide and 2466 nucleotide transcript variant sequences were submitted to GenBank and have been assigned the accession numbers: Bos taurus microRNA pri-miR-21, transcript variant 1 (MF966934), Bos taurus microRNA pri-miR-21, transcript variant 2 (MF966935).

### The proximal promoter region and transcription start site for *pri-miR-21* lie within a conserved intronic region of VMP1

Although evidence for specific factor dependent regulators of miR-21 transcription has increased recently, it has primarily been derived from studies in human cell lines and mouse models. No promoter analysis studies of the *MIR21* gene have been published to date in the cow. We therefore sought to compare the bovine promoter region predicted by our analysis of the transcription start site (TSS) to previously published data on the human promoter^47^, and to the region of conserved sequence in the mouse. A region of the bovine reference genome UMD 3.1.1 containing full length *pri-miR-21* and 1 kilobase upstream of the site TSS was used as the template to search for evolutionarily conserved regions between the cow, mouse and human genomes. As expected, the most highly conserved region among the three aligned genomes represents mature miR-21 (100% conservation) (Fig. 2C). The 72 bases representing the *MIR21* hairpin is also highly conserved. The total aligned region contains intron 10–11, exon 11, intron 11–12, exon 12 and the 3′ untranslated region of the conserved *VMP1* gene found in all 3 species. Across the aligned sequences, the second-most highly conserved region occurs, immediately upstream of the *pri-miR-21* TSS, which lies within *VMP1* intron 10–11. This region of the aligned sequences has a much higher proportion of conserved bases than protein coding exons 11 and 12 of *VMP1*, the 3′ untranslated region of VMP1, or intron 11–12, which is excluded by splicing in the *pri-miR-21* transcript variant 2 (Fig. 2A) and shows minimal conservation between species (Fig. 2C). The highly conserved region in *VMP1* intron 10–11 is described for the remainder of this text as *Bos taurus* miRNA proximal promoter region (miPPR)-21. Importantly, although the *pri-miR-21* promoter and TSS occur within *VMP1*, previous studies including an investigation in granulosa cells have shown that *VMP1* is expressed independently of *pri-miR-21* and mature *miR-21*^32,47^. We therefore consider miPPR-21 to be the major regulatory region driving *pri-miR-21* transcription.

### MiRNA proximal promoter region 21 contains two conserved binding sites for signal transducer and activator of transcription 3 (STAT3)

**Figure 3:**
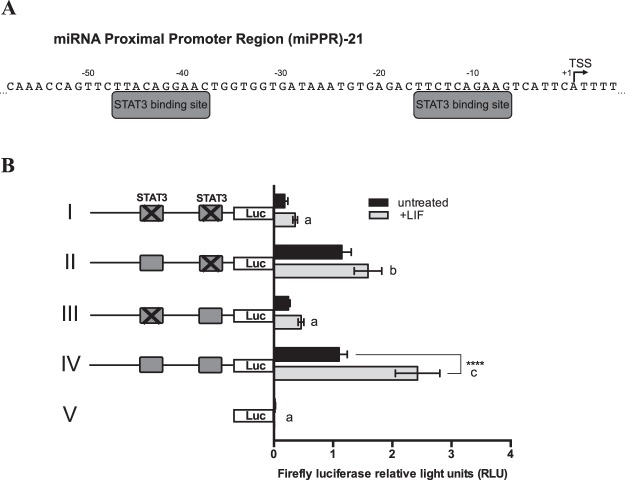
Two functional STAT3 binding sites are present on miPPR21. (**A**) Putative STAT3 binding sites from −47 to −38 positions and from −16 to −7 positions upstream of *pri-miR-21* transcription start site (TSS) (+1) on miPPR-21. (**B**) Promoter reporter assay of a Firefly luciferase vector driven by bovine miPPR-21 (pGL3miR21-Luc) containing the two STAT3 binding sites shown in (**A**). Constructs containing mutations in both binding sites (I), a single binding site (II, III), or wild-type (IV) were co-transfected into MCF7 cells with a Renilla luciferase construct for normalization. The pGL3-Luc base construct containing no promoter element (V) was also transfected as a control. Grey rectangles indicate STAT3 binding sites, and X indicates a mutation in that binding site. MCF7 cells were treated with 15 ng/mL recombinant human LIF, and luciferase activity in untreated and LIF-treated cells is shown relative to baseline activity of wild-type construct (IV) in untreated conditions. Firefly luciferase light units normalized to Renilla luciferase light units are shown. Asterisks indicate significant differences between untreated and LIF treated cells transfected with the same construct. Constructs sharing same letters did not show significant differences in luciferase activity under the influence of LIF.

STAT3 is a potent transcriptional regulator in gp130 cytokine signaling pathways, which is important in late follicle development and during oocyte maturation, the period during which miR-21 expression was seen to increase (Fig. 1A,B). Furthermore, the IL-6/gp130 pathway has been found to induce miR-21 via STAT3-mediated mechanisms in human myeloma cells^48^. We therefore postulated that STAT3 participates in *pri-miR-21* transcription in the cow and examined the miPPR-21 sequence for the presence of STAT3 transcription factor binding site consensus sequences. *In silico* analysis revealed putative sites at 16 and 47 nucleotides upstream of the transcription start site (Fig. 3A). We next generated a promoter/reporter construct consisting of a 411 nucleotide fragment of the miPPR-21 containing both STAT3 binding sites cloned upstream of the Firefly luciferase gene (pGL3miR21-Luc). When transfected into MCF7 cells, this construct was highly active and further responsive to treatment with 15 ng/mL LIF. Elimination of one or both of the STAT3 sites by site-directed mutagenesis, significantly decreased basal reporter activity and essentially eliminated LIF-responsiveness compared to the wild-type reporter construct (Fig. 3B). This was particularly obvious with elimination of the more distal STAT3 site.

### STAT3 inhibition during COC *in vitro* maturation reduces *pri-miR-21* transcription and disrupts cumulus expansion

**Figure 4:**
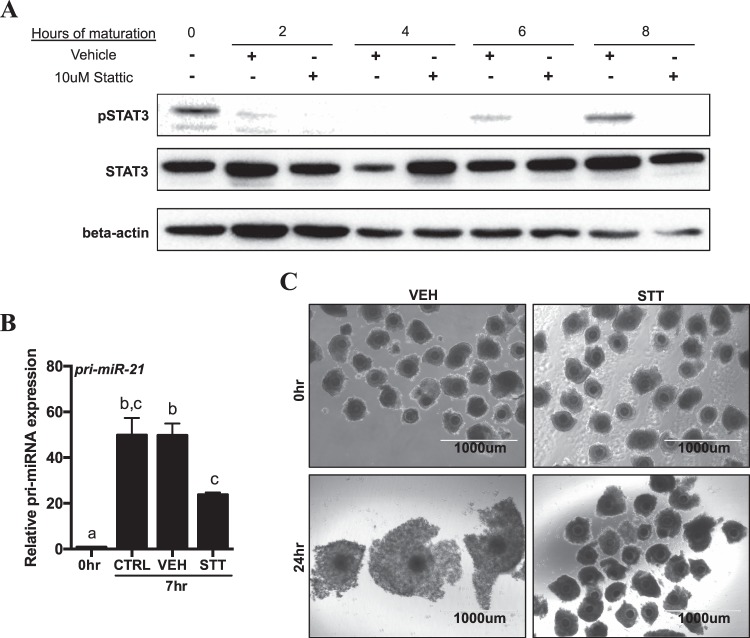
STAT3 signaling modulates the expression of *pri-miR-21* in bovine cumulus cells and STAT3 inhibition disrupts cumulus expansion *in vitro*. (**A**) Representative Western blot of STAT3 phosphorylation (Y705) and inhibition by Stattic in cumulus cells from 0–8 hr IVM. Shown with total STAT3. Beta-actin is used as a loading control. Cropped images of a single blot are shown. Full-length blots are presented in Supplemental Fig. 2. Three independent experiments were performed with similar results. (**B**) qRT-PCR of *pri-miR-21* at 0 and 7 hr IVM in cumulus cells in control media, DMSO vehicle (VEH) and 10 μM Stattic (STT). Gene expression is shown relative to the 0 hour time point. Error bars represent SEM. N = 3 biological replicates. Different letters indicate statistically significant differences between groups. (**C**) Light micrographs of immature (0 hr) and mature (24 hr) COCs matured in DMSO vehicle (VEH) or 10 μM Stattic (STT). Images obtained with EVOS FL Cell Imaging System using 4x objective.

In order to determine whether STAT3 activation was important for *pri-miR-21* expression in the context of oocyte maturation, we evaluated *pri-miR-21* expression in COCs relative to STAT3 activation status. Activation by tyrosine phosphorylation at residue 705 (Y705) is required for STAT3 to induce downstream changes in gene expression in response to cytokine stimulation^49^. We therefore first examined the dynamics of STAT3 activation in cumulus cells during *in vitro* maturation of COCs and then confirmed the effect of a specific small molecule inhibitor of STAT3 phosphorylation: Stattic^50^. While total STAT3 protein levels did not change, the levels of phosphorylated STAT3 changed markedly in several different stages in cumulus cells over the first 8 hours of maturation. Phospho-STAT3 was detectable immediately upon COC retrieval from follicles, consistent with a previous report of STAT3 status in granulosa cells from bovine follicles dissected from ovaries^37^, although this decreased over the first 2 hours of culture (Fig. 4A). Re-activation of STAT3 began in culture, and phosphorylated protein first became detectable at 6 hours of maturation, with the strongest pSTAT3 signal present after 8 hours of maturation (Fig. 4A). In some samples, pSTAT3 migrates as a doublet with a faint lower band, which is expected for the commercial antibody used in this study. At all culture time points where phosphorylated STAT3 could be detected, phosphorylation was completely inhibited by Stattic. Based on the observation that STAT3 activation was robust after 6 hours of IVM, we evaluated *pri-miR-21* transcript abundance in cumulus cells of COCs removed from culture at 7 hours of maturation (thus allowing sufficient time to observe the resultant transcriptional response) under standard conditions, supplemented with 10 μM Stattic or dimethyl sulfoxide (DMSO) vehicle. Under all conditions, *pri-miR-21* showed increased abundance compared to the 0 hour time point, however *pri-miR-21* induction was decreased by over 50% in the presence of Stattic (Fig. 4B). Importantly, after 24 hours of *in vitro* maturation COCs cultured in maturation media supplemented with Stattic also fail to undergo cumulus expansion compared to untreated controls (Fig. 4C). No detectable oocyte STAT3 phosphorylation was evident by Western blot (data not shown) despite abundant STAT3 protein in the oocyte, which is consistent with previous studies that suggest STAT3 is not activated in the oocyte^51^. We therefore focused our remaining studies on STAT3 mediated transcriptional activation on the cumulus cell compartment.

### miR-21 induction in COCs is independent of cumulus expansion, and is regulated by factors synthesized by the COC itself

**Figure 5:**
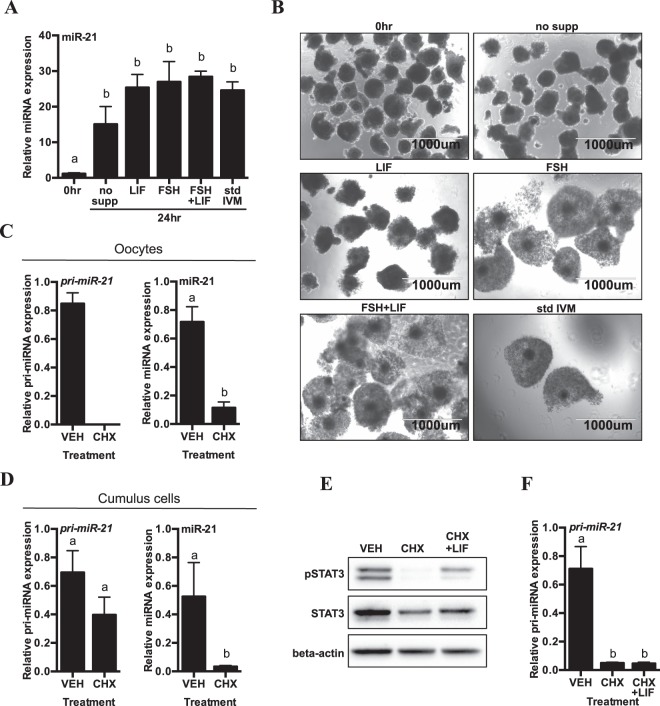
miR-21 is induced in bovine COCs over 24 hours in culture with or without serum and hormone supplementation but is sensitive to inhibition of protein synthesis. (**A**) qRT-PCR of miR-21 in cumulus cells of COCs at 0 hr or 24 hr IVM culture in serum free conditions with no growth or hormone supplementation (no supp), LIF alone, FSH alone, or FSH + LIF, as compared to standard IVM media (std IVM) which is further supplemented with LH, estradiol and FBS. Gene expression is shown relative to the 0 hour time point. Error bars represent SEM. N = 3 biological replicates. Different letters indicate statistically significant differences between groups. (**B**) Light micrographs of immature COCs (0 hr) and COCs matured *in vitro* under the same conditions described in (**A**). Images obtained with EVOS FL Cell Imaging System using 4x objective. (**C**,**D**) qRT-PCR quantification of *pri-miR-21* transcripts and mature miR-21 in (**C**) oocytes and (**D**) cumulus cells after 24 hr culture in standard IVM media containing ethanol vehicle (VEH) or cycloheximide (CHX). Gene expression is shown relative to VEH. Error bars represent SEM. N = 3 biological replicates. Different letters indicate statistically significant differences between groups. (**E**) Representative Western blot of STAT3 phosphorylation (Y705) in cumulus cells at 7 hr IVM, in serum free, non-supplemented media containing ethanol vehicle (VEH), cycloheximide (CHX) or CHX + LIF. Shown with total STAT3. Beta-actin is used as a loading control. Cropped images of a single blot are shown. Full-length blots are presented in Supplemental Fig. 3. Three independent experiments were performed with similar results. (**F**) qRT-PCR quantification of *pri-miR-21* transcripts in cumulus cells after 24 hr culture in serum free, non-supplemented media as described in (**E**). Gene expression is shown relative to VEH. Error bars represent SEM. N = 3 biological replicates. Different letters indicate statistically significant differences between groups.

To determine whether the observed changes in miR-21 expression occur as a result of the introduction of hormones or serum supplementation during culture *in vitro*, miR-21 expression was re-evaluated in the cumulus cells of COCs after 24 hours in culture under serum free conditions, without supplements, and compared to cultures supplemented with FSH alone, LIF alone, or FSH and LIF, and also with standard IVM conditions in media containing 2% FBS and supplemented with LH, FSH and estradiol. As shown in Fig. 5A, miR-21 was induced under all conditions, and increased significantly over 24 h of *in vitro* culture, including serum free culture without the supplementation of hormones or growth factors. This induction occurred independently from cumulus expansion, as COCs cultured without serum in the absence of supplements did not expand, and COCs cultured serum free in the presence of LIF expanded only slightly (Fig. 5B).

The induction of miR-21 under basal (serum-, hormone- and growth factor-free) culture conditions in cumulus cells after COC removal from the ovary suggested that ligands synthesized by cumulus cells or the oocyte during *in vitro* maturation induced miR-21 expression through autocrine or paracrine signaling. To address this possibility, *de novo* protein synthesis was inhibited by treatment with cycloheximide, as described^8,52^. In oocytes treated with cycloheximide, *pri-miR-21* was completely undetectable and the expression of mature miR-21 was also prevented (Fig. 5C). In the corresponding cumulus cells, cycloheximide prevented the expression of mature miR-21 while no significant effects were noted on the abundance of *pri-miR-21* transcripts (Fig. 5D), consistent with a transcriptional response that does not require the synthesis of additional protein factors. Cycloheximide also inhibited the STAT3 phosphorylation that is normally observed in cumulus cells after 7 hours of IVM in the absence of serum, hormone, and growth factor supplementation (Fig. 5E) suggesting that this requires synthesis of an endogenous protein ligand, receptor, or signaling factor. The importance of the synthesis of an endogenous factor in the control of *pri-miR-21* expression under these conditions is reflected in the marked reduction of *pri-miR-21* expression under minimally supplemented culture conditions in contrast to the absence of suppression in the presence of serum (Fig. 5D,F).

Ligands such as LIF that activate the Janus kinase (JAK)/STAT pathway are known to be synthesized in cumulus cells during the first 6 hours of IVM in the mouse, where they are capable of activating autocrine regulatory loops^45^. To determine whether LIF can induce phosphorylation of STAT3 in the absence of serum, hormones, and other growth factors, recombinant LIF was added to the minimal culture condition in the presence of cycloheximide. The addition of exogenous LIF to serum and hormone free culture media supplemented with cycloheximide was sufficient to activate STAT3 phosphorylation (Fig. 5E), however LIF-driven STAT3 activation alone was not sufficient to fully restore *pri-miR-21* expression in the presence of cycloheximide in the serum free conditions (Fig. 5F).

## Discussion

This study characterizes the dynamics of miR-21 expression and reveals STAT3 as a transcriptional activator of miR-21 in the cumulus compartment of the bovine cumulus oocyte complex. *Pri-miR-21* transcription is induced in cumulus cells over the first 7 hours of maturation while the mature form of miR-21 does not peak until 24 hours of IVM, which is consistent with active miRNA transcription and processing by the Microprocessor complex. The RNaseIII Drosha forms the enzymatic core of this complex, and has recently been shown to be expressed in cumulus cells^53^. *Pri-miR-21* transcripts as well as mature miR-21 also accumulate in the oocyte over this time period, though mRNA transcription generally decreases in fully-grown GV oocytes and largely ceases after germinal vesicle breakdown (GVBD)^54,55^. The accumulation of miR-21 in the oocyte is likely the result of multiple factors. Some transcriptional activity in the oocyte, has been shown to occur for specific transcripts that may contribute to the events of oocyte maturation and early embryo development^56^, and transport of precursor and mature miRNA from cumulus cells to the oocyte during IVM via transzonal projections has also been suggested^57^. The possibility of miRNA transport from cumulus cells to the oocyte would be consistent with well-established roles for cumulus cells in providing factors to the oocyte that facilitate the acquisition of developmental competence, and demonstrates how ligands that signal through STAT3 have the potential to influence gene expression in the oocyte without activation of STAT3 protein in the oocyte itself.

Physical interactions between activated STAT3 and its cognate binding sites in the human miR-21 promoter have been demonstrated previously^58^. Here we show that the *Bos taurus* miR-21 promoter contains two functional STAT3 binding sites, and that promoter activity in response to the gp130 cytokine LIF is significantly reduced when these binding sites are mutated. *Pri-miR-21* transcription is reduced in cumulus cells when COCs are cultured in the presence of an inhibitor of STAT3 phosphorylation, which markedly decreases cytokine-induced miR-21 transcription in other models^59^.

In our model of *in vitro* oocyte maturation, the increase in *pri-miR-21* over the initial 7 hours of IVM coincides strongly with the induction of key cumulus expansion related genes *Has2* and *Tnfaip6*, identified in previous bovine studies^60,61^ and confirmed here. *Has2* and *Tnfaip6* (which is induced significantly by 7 hours and undergoes a further increase throughout IVM) are involved in the formation of the hyaluronan-rich cumulus extracellular matrix^14,62^ and are among a subset of genes induced during cumulus expansion that are associated with inflammation. Ovulation has many characteristics typical of an inflammatory response^63^, and a number of genes previously considered to be limited to immune cells have been found in follicular cells^64,65^ (thoroughly reviewed by^66^). These cells are also capable of secreting and responding to inflammatory cytokines, a number of which can be detected in follicular fluid^67^. Evidence is mounting that cytokines play important roles at the peri-ovulatory period *in vivo* and during the final maturation of cumulus-oocyte complexes matured *in vitro*^64^ (see review by^68^. A number of genes upregulated during FSH-induced COC expansion *in vitro* including *Prostaglandin-endoperoxide synthase 2* *(Ptgs2)*, *Has2*, *Tnfaip6*, and *Pentraxin-related protein* *(Ptx3)* are induced during IVM by STAT3 activating cytokines^45^.

LIF is a member of the gp130-binding family of cytokines that also includes IL-6, IL-11 and oncostatin M (OSM) which share the common membrane receptor and signal transducer gp130^69,70^. A structurally similar membrane receptor to gp130 (leptin receptor, OB-R) is activated by the hormone leptin and also results in phosphorylation of STAT3 and the induction of responsive genes^71^. At the mRNA level, our data clearly demonstrate that *Lif* is strongly induced in cumulus cells during oocyte maturation in the cow, and that LIF alone can partially induce cumulus expansion under minimal culture conditions. This contrasts to some extent with findings in the mouse, where IL-6 appears to be the predominant gp130 binding cytokine. While *Il6* is clearly present, *Lif* was strongly induced during *in vitro* maturation, prompting us to focus on its potential effects in this system. This is clearly relevant to species differences likely to exist between cattle and mice with respect to the predominant gp130 cytokines, since overlapping functions of these cytokines are likely to result in largely conserved outcomes in this cellular context.

The LIF/LIF-receptor complex (gp130 plus the ligand-specific receptor) is expressed in bovine cumulus cells^72^ and supplementation with LIF benefits oocyte maturation, cumulus expansion and embryo development in humans, mice and domestic animal species^44,73–75^. Bovine COCs matured with the addition of LIF to *in vitro* culture media showed a significant increase in the number of oocytes reaching metaphase II as well as enhanced cytoplasmic oocyte maturation, as indicated by a greater proportion of oocytes showing cortical granule distribution consistent with oocyte competency^73^. IL-6 and leptin are clearly also important in many aspects of follicular development and *in vitro* oocyte maturation, and may have similar effects here through their common effects on STAT3 activation. Importantly, evidence shows that LIF, IL-6, and leptin are all capable of inducing miR-21^48,58,59,76^ in different cell types. As miR-21 induction is the primary focus of the current study, we chose to employ this ligand as a relevant activator of STAT3 in the context of bovine cumulus and oocyte biology.

STAT3 is a critical signaling molecule and transcriptional activator in the ovary, particularly in the peri-ovulatory period and during *in vitro* maturation. The present study provides clear evidence of a specific role for STAT3 in the induction of miR-21 in bovine cumulus cells, however this analysis would be incomplete without a discussion of the additional signaling cascades that are likely contribute to miR-21 induction during IVM. Treatment with Stattic reduced *pri-miR-21* accumulation by ~50% compared to vehicle controls, however *pri-miR-21* still increased significantly from the 0 hour time point.

While we have clearly shown that STAT3 activation induces *pri-miR-21* transcription during cumulus expansion and oocyte maturation, it should be emphasized here that other signaling pathways are also likely to be important in this highly conserved cellular response. Redundant and synergistic activation is likely by factors such as amphiregulin^77^ acting through multiple intracellular signaling cascades including STAT3, Ras/Raf/MAPK, ERK1/2 and activator protein 1 (AP-1) family transcription factors^78^ which can initiate *pri-miR-21* transcription^47^. Furthermore, gp130 cytokines can also act through these other pathways to reinforce signaling responses^73,79,80^. Moreover, a recent report suggests that miR-21 can also be induced by oocyte-secreted factors such as Growth-differentiation factor 9 (GDF9)^53^. The potential for these pathways to activate *pri-miR-21* transcription is strongly supported by the presence of multiple distinct *cis-*acting elements in the cloned promoter region (Supplemental Table 1). The presence of multiple pathways which are capable of inducing this important miRNA highlight its potential importance in oocyte and cumulus cell biology.

While the actual combination of ligands that normally induce miR-21 via JAK/STAT signaling in the cumulus oophorus remain to be determined, the data presented here and the studies discussed above demonstrate that COCs synthesize one or more ligands capable of initiating signaling cascades that increase miR-21 expression. Our data reveal that, under standard culture conditions, cycloheximide treatment abrogates miR-21 expression but does not significantly impair *pri-miR-21* transcription in cumulus cells, indicating that the processing of precursor miRNAs into mature forms requires protein synthesis but the transcriptional response does not, and may be initiated by factors normally present in serum or media. When protein synthesis is inhibited under serum- and hormone-free conditions *pri-miR-21* transcription is essentially absent and STAT3 phosphorylation is markedly reduced, strongly supporting the physiological relevance of the STAT3 transcriptional activation identified in our reporter studies. Importantly however, since the addition of exogenous LIF does not restore *pri-miR-21* transcription in cycloheximide-treated COCs, it is likely that the synthesis of additional protein participants in the signaling pathways is necessary for *pri-miR-21* induction in maturing COCs. Together with the literature discussed above, our results suggest that cumulus cell miR-21 induction is likely a product of both paracrine and autocrine signaling in the COCs for which STAT3 activation is necessary but not sufficient.

The physiologic roles that miR-21 may play in the control of cumulus cell gene expression and function are likely to be complex and lie outside the scope of this study. miR-21 has been widely implicated in apoptosis and cellular proliferation through the modulation of cellular factors such as programmed cell death 4 (PDCD4)^81^ and phosphatase and tensin homolog (PTEN)^82^, both of which are important in granulosa cell and oocyte biology^33,83,84^. While an increased miR-21 clearly accompanies normal oocyte maturation, some clinical studies have shown that abnormally elevated miR-21 may actually correlate with impaired fertility^85^. In cattle, elevated miR-21 expression correlates with large atretic follicles when compared to large healthy follicles^86^. Notably, follicular atresia in cattle also correlates with enhanced LIF/STAT3 signaling in granulosa cells^87^, which may reflect a functional association in this context. While miR-21 inhibition induces apoptosis in ovarian cells and reduces ovulation^32,53^, elevated miR-21 is associated with follicular atresia, poor ovarian response and inflammatory disease. It is therefore likely that an optimal level of miR-21 is necessary for a healthy periovulatory follicle, and for COCs that mature successfully *in vitro*. The function and overall importance of miR-21 in normal follicle development and *in vitro* maturation, whether expressed via STAT3 dependent or independent pathways clearly warrants further mechanistic investigation.

In summary, the data presented here have clearly shown that miR-21 expression during bovine oocyte maturation and cumulus expansion is influenced by STAT3 signaling. This study further suggests that additional pathways in the ovary are likely to contribute to this response. Identification of additional normal pathways and those stimulated by pathophysiologic processes leading to miR-21 expression in the complex follicular environment should substantially enhance our understanding of the roles it plays in normal and abnormal ovarian biology and fertility.

## Methods

### Cumulus oocyte complex collection and *in vitro* maturation

Bovine ovaries were obtained from a local abattoir (Cargill, Guelph, ON) and transported at 35 °C. Within 2 hours of ovary collection, cumulus-oocyte complexes (COC) were aspirated from follicles greater than 6 mm diameter using vacuum aspiration. Collected complexes were placed into 1 M HEPES-buffered Nutrient Mixture F-10 Ham (Sigma-Aldrich, St. Louis MO) collection media supplemented with 2% fetal bovine serum (FBS) (Thermo Fisher Scientific, Waltham, MA), Hepalene (2 IU/ml, LEO Pharma Inc, Thornhill, ON), 14.3 mM sodium bicarbonate (Sigma-Aldrich) and penicillin 50 IU/mL/streptomycin 50 IU/mL (Invitrogen, Burlington, ON).

COCs were washed twice in 1M HEPES-buffered TCM-199 maturation medium (Caisson Labs, North Logan UT) supplemented with 22 μg/mL sodium pyruvate (Sigma-Aldrich) and penicillin 50 IU/mL/streptomycin 50 IU/mL (Invitrogen). Oocytes for maturation were placed in groups of 30–40 in 400 μL maturation medium containing 0.5 μg/mL FSH, 1 μg/mL LH and 1 μg/mL estradiol (National Institutes of Health (NIH), USA) in a humidified atmosphere at 38.5 °C and 5% CO_2_. After 7 or 24 hours, COCs were denuded and collected. At the time of collection, COCs were washed twice in phosphate buffered saline (PBS) with 0.1% polyvinyl alcohol (PVA) (Sigma-Aldrich) and cumulus cells were separated by physical disruption in PBS/PVA. Cumulus cells were placed into 1.5 mL microcentrifuge tubes and centrifuged at 600 × g for 6 minutes, and PBS/PVA was carefully removed from pellets. After cumulus cell collection, denuded oocytes were treated with 2 mg/mL Hyaluronidase from *Streptomyces hyalurolyticus* (Sigma-Aldrich) to remove any remaining cumulus cells, and washed in PBS with 0.1% PVA. Upon collection, all samples were immediately flash frozen in liquid nitrogen and stored at −80 °C.

For specific experiments, IVM medium was supplemented with 25 μg/mL cycloheximide^8^ from microbial sources (Sigma-Aldrich) dissolved in 100% ethanol, or 10 μM Stattic (Selleckchem, Houston TX) dissolved in dimethyl sulfoxide (DMSO) (Sigma-Aldrich). COC collection and maturation was modified as follows for serum-free experiments: COCs were aspirated and collected in serum-free 1 M HEPES-buffered TCM-199 (Sigma-Aldrich) collection media with 2 IU/ml Hepalene (LEO Pharma), penicillin 50 IU/mL/streptomycin 50 IU/mL (Invitrogen) and 0.1% PVA, followed by *in vitro* culture in 1 M HEPES-buffered TCM-199 maturation medium (Sigma-Aldrich) supplemented with 22 μg/mL sodium pyruvate (Sigma-Aldrich), penicillin 50 IU/mL/streptomycin 50 IU/mL (Invitrogen) and 0.1% PVA. IVM was then supplemented with 25 ng/mL recombinant human LIF (R&D Systems, Minneapolis MN) or 0.5 μg/mL FSH (NIH). In studies where a number of treatment groups and conditions were required, it was not always possible to collect multiple samples of each condition at a single collection event. In order to appropriately analyze data under these circumstances, at least one complete set of samples from all experimental conditions were collected, and each resulting data value was divided by the average of the entire sample set, thus standardizing all trials before comparing differences between experimental conditions. In the case of protein quantification and Western blotting, all samples that appear on blots together were collected during a single experimental trial and are compared only to other experimental conditions of that same trial.

### RNA isolation and quantitative PCR

Total RNA, including small RNA, was isolated using the miRNeasy Micro kit (Qiagen, Mississauga, ON) according to the manufacturer’s protocol, and with the inclusion of DNase digestion performed on-column with the RNase-free DNase Set (Qiagen). RNA was eluted in nuclease-free water and RNA quantified by Nanodrop 2000c (Thermo Fisher). RNA concentrations ranged from approximately 20–50 ng/uL. Total cellular RNA (containing pri-miRNAs) was isolated from oocytes using the PicoPure RNA isolation kit (Thermo Fisher) with on-column DNase digestion (Qiagen), as reproducible detection of these infrequent transcripts could not be achieved when RNA was isolated using the previously mentioned system. RNA was isolated from pools of 30 COCs as cumulus or oocyte fractions for use with miRNeasy kit, and from pools of 5 oocytes for use with PicoPure kit. Messenger RNA and pri-miRNA were reverse transcribed with qScript complementary DNA (cDNA) SuperMix (Quantabio, Beverly MA), and miRNA was extended by polyadenylation then reverse transcribed with qScript microRNA cDNA Synthesis Kit (Quantabio). qRT-PCR was performed with a CFX96 Touch Real-Time PCR Detection System (BioRad Laboratories, Inc., Hercules, CA) using PerfeCTa SYBR Green SuperMix (Quantabio). cDNAs encoding pri-miRNAs and mRNAs were amplified using specific forward and reverse primers (Table 1), while miRNAs were amplified with a gene-specific forward primer and PerfeCTa Universal PCR Primer (Quantabio). Three ng of cDNA template was used for each reaction. Efficiencies were calculated by standard curve for all primers designed in this study and gene expression was calculated by the efficiency-corrected ΔΔCt method^88^. Cumulus cell mRNA and pri-miRNA quantification were normalized to the average of reference genes *Tyrosine 3-monooygenase/tryptophan 5-monooxygenase activation protein zeta (YWHAZ)*^89^ and *Beta-actin* *(ACTB)*, which were determined to be the most stable using the geNorm algorithm^90^. Oocyte pri-miRNA quantification was normalized to *YWHAZ* and *Glyceraldehyde-3-phosphate dehydrogenase (GAPDH)*, which was found to be more stable in these samples. miRNA were normalized to snRNA U6, which has been shown previously to be suitable for oocytes^27^, and is stably expressed in cumulus cells. Expression of each gene is shown relative to the abundance of that gene in the control group for a given experiment, and so relative abundance and changes in gene expression are comparable only within an experiment and not between experiments.

**Table 1:** Genes and specific primer sequences used in quantitative RT-PCR.

Gene symbol	Gene name	NCBI Reference Sequence	Primer set sequences (5′-3′)	Product length (nt)	Reference
ACTB	actin beta	NM_173979	F: CCTTCCTGGGCATGGAATCCT R: TCTTCATTGTGCTGGGTGCC	186	*
AREG	amphiregulin	NM_001099092	F: ACTTTGGTGAACGATGTGGGG R: TCGTCTTCGAAGCAGGATTGTA	155	*
GAPDH	glyceraldehyde-3-phosphate dehydrogenase	NM_001034034	F: TGTTGTGGATCTGACCTGCC R: TGTCGTACCAGGAAATGAGCTT	224	*
HAS2	hyaluronan synthase 2	NM_174079	F: TAAATGTGGCAGGCGGAAGAAGG R: GTCTTTGTTCAAGTCCCAGCAGCA	183	^60^
IL6	interleukin 6	NM_173923	F: CAATCTGGGTTCAATCAGGCGAT R: GCATCTTCTCCAGCAGGTCAG	220	*
LIF	leukemia inhibitory factor	NM_173931	F: TCCTCTATTACACGGCCCAGG R: TCACGTGGTACTTGCTGCAC	294	*
pri-miR-21	primary-miR-21	MF966934 MF966935	F: ATGGCTGTACCACCTTGTCG R: GTGCCACTAGACCTAAGGACC	192	*
STAT3	signal transducer and activator of transcription 3	NM_001012671	F: CTCTCCCCACTTCTGCCAAG R: AGGGGTCACAACTGCTGCTC	118	*
TNFAIP6	TNF alpha induced protein 6	NM_001007813	F: TGTCCTGCTATGGGAAGAGG R: TGCTTGTAGGTGGCAAGATG	186	^61^
YWHAZ	tyrosine 3-monooygenase/tryptophan 5-monooxygenase activation protein zeta	BM446307 XM_019973801	F: GCATCCCACAGACTATTTCC R: GCAAAGACAATGACAGACCA	120	^89^

*This study.

### miRNA *in situ* hybridization

All chemicals used for the *in situ* hybridization came from Sigma-Aldrich unless otherwise stated. Slides containing sections of mature COCs were deparaffinised in xylene and ethanol before digestion with Proteinase-K for 10 minutes at 37 °C in a humidified chamber. Sections were then fixed in 4% formaldehyde (Thermo Fisher) for 10 min at room temperature, then rinsed twice in a solution of 0.13 M 1-methylimidazole and 300 mM sodium chloride pH 8.0 and incubated in 0.16 M l-ethyl-3- (3-dimethylaminopropyl) carbodiimide (EDC)/0.13 M 1-methylimidazole/NaCl for 1 hr at room temperature. Slides were then washed in 0.2% (w/v) glycine/TBS and treated with 1% hydrogen peroxide to block endogenous peroxidases, followed by dehydration in ethanol before hybridization. Double digoxigenin (DIG) LNA modified probes (Exiqon, Copenhagen, Denmark) were denatured at 90 °C for 4 min and applied to slide surface at the following concentrations: 1 nM U6 snRNA probe (5′-CACGAATTTGCGTGTCATCCTT-3′), 40 nM miR-21 probe (5′-TCAACATCAGTCTGATAAGCTA-3′), or 40 nM scramble probe (5′-GTGTAACACGTCTATACGCCCA-3′), and placed in a humidified hybridizing chamber in a 55 °C oven for 1 hr. Slides were then washed in saline-sodium citrate at hybridization temperature, and blocked in PBS with 2% sheep serum (Thermo Fisher), 1% BSA and 0.1% Tween. Anti-DIG reagent (sheep anti-DIG POD-conjugate (Roche, Indianapolis IN) was applied for 1 hr, followed by several washes in PBS before incubation with TSA-PLUS_Cy3 according to the manufacturer recommendations (TSA plus cyanine 3 system, PerkinElmer, Waltham MA) in the dark for 10 min at room temperature. After several washes in PBS, slides were counterstained with 300 nM 4′,6-Diamidino-2-phenylindole dihydrochloride (DAPI) in PBS for 2 min at room temperature, rinsed multiple times in PBS and mounted with coverslips using Prolong Gold Antifade (Thermo Fisher) and cured for 24 hr before imaging.

### Equipment and settings

*In situ* hybridized slides were imaged (16 bit) using a confocal microscope LSM 700 (Zeiss) with a 40×/0.95 objective at 555 nm for Cy3 and 405 nm for DAPI. Zen Black 2012 software (Zeiss) and ImageJ (NIH) were used to process all images. DAPI acquisitions were performed with detector gain at 520, with excitation using a 405 nm laser at 2%, and emission with a dichroic beam splitter at 582 nm with a low pass. Cy3 acquisitions were performed with detector gain at 350, with excitation using a 555 nm laser at 2% and emission with a dichroic beam splitter at 559 nm with a high pass. All images were acquired using the same setting. Live cell images of COCs were obtained with EVOS FL Cell Imaging System using a 4x objective.

### 5′ and 3′ RACE PCR analysis and full length *pri-miR-21* cloning

The transcription start site and polyadenylation signal of the primary transcript of miR-21 were identified by 5′ and 3′ rapid amplification of cDNA ends (RACE). Total RNA was isolated from bovine testis using miRNeasy kit (Qiagen). 5′ RNA ligase mediated (RLM)-RACE and 3′RACE were performed with GeneRacer kit (Invitrogen). For 5′RACE, 5 μg RNA was dephosphorylated with calf intestinal phosphatase (CIP), followed by phenol:chloroform extraction before RNA precipitation. RNA pellets were air dried and re-solubilized in nuclease-free water for treatment with tobacco acid pyrophosphatase (TAP), then re-extracted and dried as above. RNA was 5′ ligated to adapters and reverse transcribed with random primers and Superscript III, followed by treatment with RNaseH. RT-PCR reactions were carried out using a RACE adapter primer and a 5′RACE gene specific primer (Table 2), using Platinum Taq HiFi polymerase (Invitrogen), according to manufacturer’s instructions. 5′ ends of *pri-miR-21* were amplified by touchdown PCR method for a total of 35 cycles according to the following protocol: Initial denaturation at 94 °C for 2 min, followed by 6 cycles of 94 °C × 30 s and 72 °C × 2 min, followed by 6 cycles of 94 °C × 30 s and 70 °C × 2 min, followed by 23 cycles of 94 °C × 30 s, 65 °C × 30 s and 68 °C × 2 min, followed by a final extension at 68 °C for 10 min. For 3′RACE, 1 μg testis RNA was reverse transcribed with GeneRacer Oligo dT primer and Superscript III after which RT-PCR was carried out as above (see Table 2 for 3′RACE gene specific primer). cDNAs of multiple lengths were separated on 1.5% agarose gels and extracted using PureLink Quick Gel Extraction kit (Invitrogen). Sequences of cDNA ends were obtained by cloning into pGEM-T Easy vector (Promega, Madison, WI) for sequencing, according to the manufacturer’s instructions. Briefly, RACE-PCR products were ligated at 4 °C overnight into linearized vector with a single 3′-terminal thymidine overhang at each end. Circular DNA was then transformed into high-efficiency DH5α competent cells and plated onto lysogeny broth (LB) (Sigma-Aldrich) agar plates with Ampicillin/IPTG/X-Gal (Sigma-Aldrich/Thermo Fisher/Promega) for overnight culture at 37 °C. Transformants were assessed for successful cloning inserts by blue:white screening and positive colonies were selected for liquid culture in LB with Ampicillin. Plasmid DNA was purified by Miniprep (Qiagen) according to manufacturer’s instructions. The sequences and orientation of inserts were confirmed by Sanger sequencing (Laboratory Services, University of Guelph). 5′ and 3′ RACE product sequences were used to design oligonucleotides to amplify the full length primary miR-21 transcript (Table 2) from the bovine testis template used for 3′ RACE, according to the touchdown PCR protocol described above. No template control (NTC) reactions were run alongside templates to ensure that all PCR reagents were free from DNA contamination. Transcript variants were extracted from agarose gels, cloned into pGEM-T Easy and confirmed by sequencing.

**Table 2:** Specific primer sequences used for RACE PCR, cloning and site directed mutagenesis.

Primer name	Primer sequence (5′-3′)
RACE	
pri-miR-21 5′RACE Gene Specific Primer pri-miR-21 3′RACE Gene Specific Primer	GCTGCTGGATTCGTTTGGCGT TCATGGCAACAGCAGTCGATGG
pri-miR-21 Cloning	
pri-miR-21 Cloning Forward pri-miR-21 Cloning Reverse	TGAGCTCGCCTCACTCTGAGAACT AATGCGGGTGAAGGTGATGACAGAC
miPPR-21 Cloning	
miPPR-21 KpnI Forward miPPR-21 BglII Reverse	GATCGGTACCTGCGTGTTTTGAA GATCAGATCTGAATGACTTCTGAGAAG
miPPR-21 STAT3 Site Directed Mutagenesis	
miPPR-21-41GΔC-40AΔG Forward miPPR-21-41GΔC-40AΔG Reverse	GTTCAAACCAGTCCTTACAGCGACTGGTGGTGATAAATGTGA TCACATTTATCACCACCAGTCGCTGTAAGGACTGGTTTGAAC
miPPR-21-9AΔG-8AΔC Forward miPPR-21-9AΔG-8AΔC Reverse	GTGATAAATGTGAGACTTCTCAGCGGTCATTCAGATCTGCGATCTAAG CTTAGATCGCAGATCTGAATGACCGCTGAGAAGTCTCACATTTATCAC

### Promoter analysis and transcription factor binding site prediction

Using the bovine UMD3.1 assembly as a reference genome^91^, the ECR Browser^92^ tool was employed to locate regions of *pri-miR-21* and its 1 kb upstream putative regulatory region that are conserved between bovine and the human (assembly GRCh37) or mouse (assembly GRCm38) sequences. Evolutionarily conserved regions were searched for putative transcription factor binding sites in the JASPAR database^93^.

### miR-21 proximal promoter cloning and site-directed mutagenesis

Bovine DNA was isolated from bovine testis using Zygem prepGEM tissue (Zygem, Charlottesville VA) according to manufacturer’s directions. The miR-21 proximal promoter region was amplified from bovine DNA using primers specific to a region ~400 nucleotides upstream of the putative *pri-miR-21* transcription start site, using specific primers with the addition of restriction enzyme target sites for KpnI and BglII (Table 2), and cloned into pGEM-T vectors as above. To generate luciferase vectors driven by the miR-21 promoter, the most proximal 400 nucleotides upstream of the *pri-miR-21* transcription start site were subcloned into pGL3 vector (Promega). Site directed mutagenesis was performed using QuikChange II site-directed mutagenesis kit (Agilent, Santa Clara CA) according to manufacturer’s instructions, by generating a GΔC, AΔG double mutation at position −41 to −40 upstream of the *pri-miR-21* transcription start site, or a AΔG, AΔC double mutation at position −9 to −8 (see Table 2 for mutagenesis primers).

### Promoter reporter assays

7 × 10^4^ MCF7 human breast cancer cells (ATCC, Manassas, VA, Cat# CRL-12584, RRID:CVCL_0031) were seeded onto tissue culture-treated 24-well plates (Sigma-Aldrich) and maintained in 1 mL Roswell Park Memorial Institute (RPMI) 1640 Medium (Thermo Fisher) supplemented with 4 mM L-glutamine (Sigma-Aldrich), 10% FBS, 1 mM sodium pyruvate (both Thermo Fisher), and 1% penicillin/streptomycin (Sigma-Aldrich), and incubated at 37 °C with 5% CO_2_ conditioned air. Cells were cultured for 48 h after plating until they reached ~70% confluency, washed once in sterile PBS and changed to serum-free Opti-MEM media (Thermo Fisher) for serum starvation and transfection of reporter constructs. After 8 hours starvation, 450 ng Firefly-luciferase reporter construct and 50 ng pRL Renilla-luciferase control vector (Promega) were co-transfected using Lipofectamine LTX (Thermo Fisher). 1 µL Lipofectamine or 500 ng Firefly and Renilla-luciferase constructs were diluted in Opti-MEM, mixed in a 1:1 ratio and incubated for 5 min at room temperature before transfection. After 4 hours of recovery, transfected cells were cultured for 4 hours in the presence or absence of 15 ng/mL LIF, then lysed and prepared for luminescence quantification using Dual-Luciferase Reporter Assay System (Promega) according to manufacturer’s instructions. Luciferase activity was evaluated using a FLUOstar Optima microplate luminometer (BMG Labtech, Cary NC). All experiments were performed in technical duplicates and repeated a minimum of three times.

### Protein isolation and Western blotting

Cumulus cells from 40 COCs were lysed in 42uL radioimmunoprecipitation assay (RIPA) buffer (50 mM pH 7.4, 1% Triton-X 100, 0.5% sodium deoxycholate, 0.1% sodium dodecyl sulfate (SDS), 15 mM sodium chloride, 2 mM ethylenediaminetetraacetic acid (EDTA)), with phosphatase/protease inhibitor cocktail (Bimake, Houston TX) on ice. Samples were sonicated for 15 seconds, incubated on ice for 5 minutes and centrifuged at 12,000 × g for 10 minutes. Proteins were denatured by boiling in SDS protein loading buffer containing dithiotreitol (DTT), separated by gel electrophoresis on 8% polyacrylamide gels, and transferred to polyvinylidene fluoride membranes. Membranes were blocked for 1 hour in 5% BSA (Sigma-Aldrich) and incubated at 4 °C overnight in the following primary antibodies: phosphoSTAT3 (Y705) (Cat# 9145, RRID:AB_2491009) at a final dilution of 1:1000, native STAT3 (Cat# 4904, RRID: AB_331269) at 1:2000, Drosha (Cat# 3364, RRID: AB_2238644) at 1:1000 and β-actin (Cat# 4967, RRID: AB_330288) at 1:5000. Membranes were washed several times in Tris buffered saline pH 7.6 plus 0.1% Tween (TBS-T) (Sigma-Aldrich) before a 1 hr incubation with a horseradish peroxidase-conjugated secondary antibody (Cat# 7074, RRID: AB_2099233) at 1:5000. All antibodies used were from Cell Signaling Technology (Danvers, MA). Membranes were immersed in Clarity Western ECL Blotting Substrate (Bio-Rad) and imaged on a ChemiDoc XRS + Imaging system (Bio-Rad).

### Statistical analysis

Differences among groups were analyzed by ANOVA after Tukey’s multiple comparisons test, or by one-tailed t-test when the experimental design consisted of only two groups, using GraphPad Prism 6 (GraphPad Software, La Jolla CA). Gene expression data collected from qRT-PCR were log_2_ transformed in order to conduct the appropriate statistical analysis. A minimum of N = 3 biological replicates were performed for all reported data, and a p-value < 0.05 was considered statistically significant. Data shown represent the mean +/− standard error of the mean (SEM). Different letters indicate statistically significant differences between groups.

## Electronic supplementary material

Supplementary Data 1
