# Supplementary material for: STAT3 signaling stimulates miR-21 expression in bovine cumulus cells during in vitro oocyte maturation
Source: Sci Rep. 2018 Aug 1;8:11527. doi: 10.1038/s41598-018-29874-w (PMC6070548; doi:10.1038/s41598-018-29874-w)
Supplement: Supplementary file 1 — Supplementary Data 1 [file 41598_2018_29874_MOESM1_ESM.pdf]

Supplemental Data to:

STAT3 signaling stimulates miR-21 expression in bovine cumulus cells during *in vitro* oocyte maturation

Allison Tscherner (tscherner@uoguelph.ca)[1]
Alyssa Brown (abrown49@uoguelph.ca)[1]
Leanne Stalker (stalkerl@uoguelph.ca)[1]
Jennifer Kao (jenn.kao@mail.utoronto.ca)[1]
Isabelle Dufort (isabelle.dufort@fsaa.ulaval.ca)[2]
Marc-André Sirard (Marc-Andre.Sirard@fsaa.ulaval.ca)[2]
Jonathan LaMarre (jlamarre@uoguelph.ca)[1]*

Supplemental Figure 1

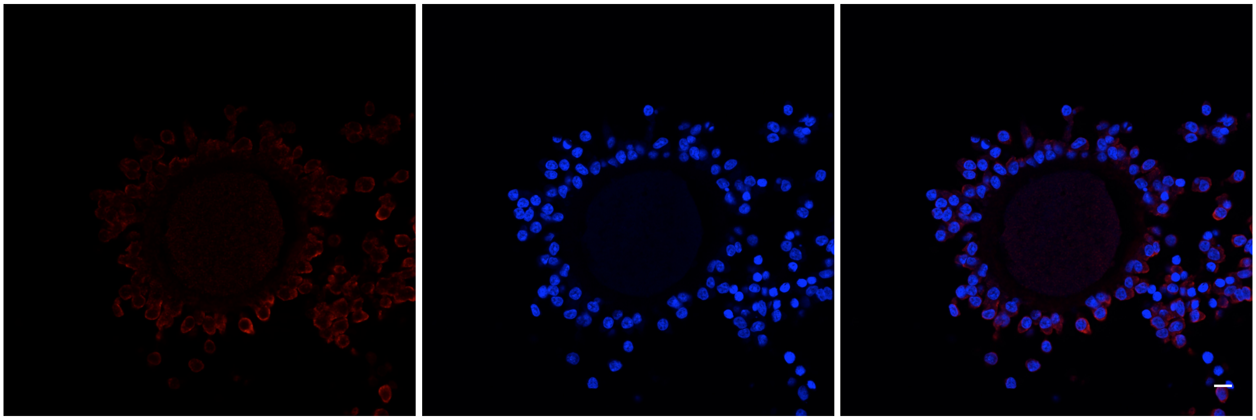

Supplemental Figure 1: RNA in situ hybridization of mature COC. Left panel: scrambled RNA probe, center panel: nuclei stained with DAPI, right panel: merged image. Scale bar represents 10µm.

Supplemental Figure 2

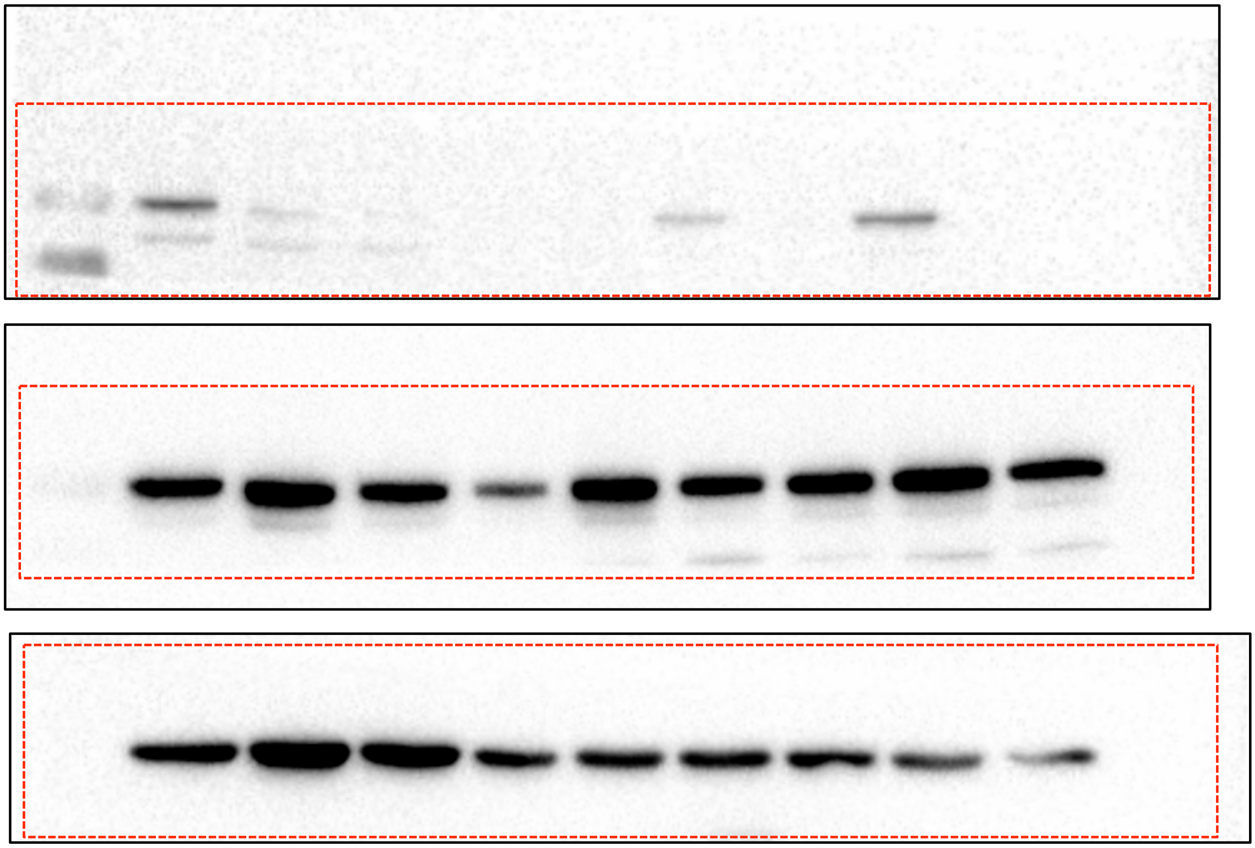

Supplemental Figure 2: Representative Western blot of STAT3 phosphorylation (Y705) and inhibition by Stattic in cumulus cells from 0-8hr IVM. Full-length blots corresponding to the cropped images presented in the main text, Figure 4. Red hatched lines delineate the approximate boundaries of the original membrane.

Supplemental Figure 3

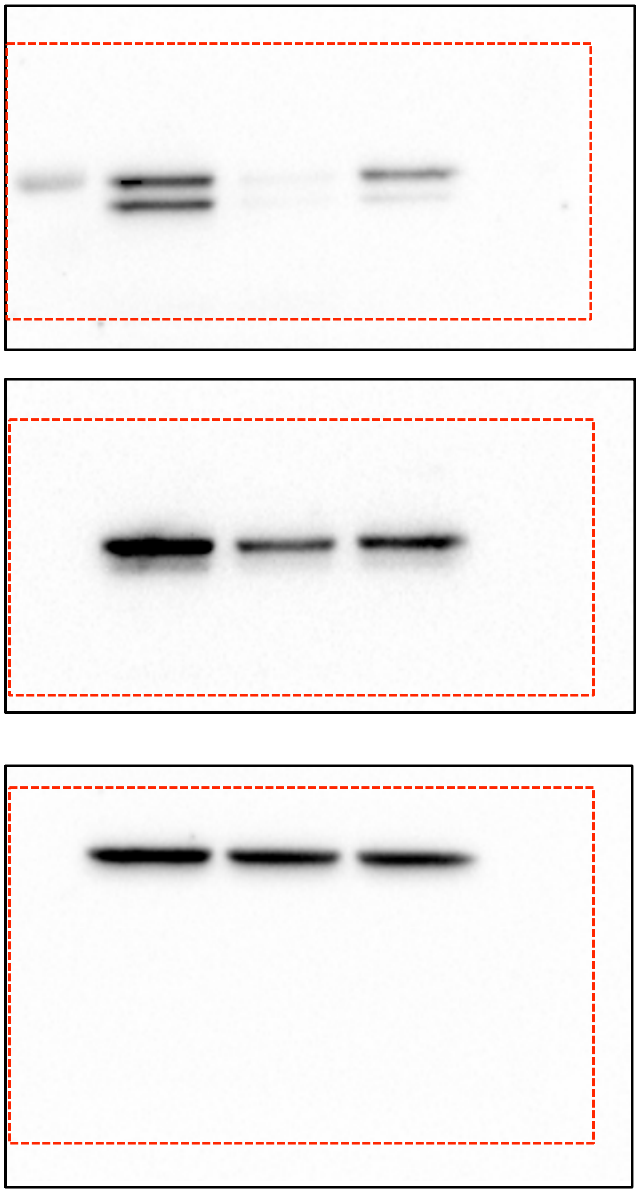

Supplemental Figure 3: Representative Western blot of STAT3 phosphorylation (Y705) in cumulus cells at 7hr IVM, in serum free, non-supplemented media containing vehicle (ethanol), cycloheximide (CHX) or CHX+LIF. Full-length blots corresponding to the cropped images presented in the main text, Figure 5. Red hatched lines delineate the approximate boundaries of the original membrane.

Supplemental Table 1: Transcription factors predicted to bind miPPR-21 on plus strand

	Bos *taurus*						Homo *sapiens*					
	Position (TSS=+1)						Position (TSS=+1)					
**TF**	**Start1**	**End1**	**Strand1**	**Score1**	**%Score1**	**Seq1**	**Start2**	**End2**	**Strand2**	**Score2**	**%Score2**	**Seq2**
**HNF4A**	-306	-294	+	8.096	80.7	GATTCAAGGTTCA	-305	-293	+	10.627	86.8	GATTCAAAGTTCA
**ARID3A**	-305	-300	+	5.399	82.7	ATTCAA	-304	-299	+	5.399	82.7	ATTCAA
**HNF4A**	-299	-287	+	8.42	81.5	GGTTCATAGTTCC	-298	-286	+	8.468	81.6	AGTTCATAGTTCC
**ELF5**	-293	-285	+	9.888	94.4	TAGTTCCTT	-292	-284	+	9.888	94.4	TAGTTCCTT
**Sox17**	-286	-278	+	6.176	82.3	TTCTTTGTT	-285	-277	+	6.176	82.3	TTCTTTGTT
**ELF5**	-282	-274	+	7.685	87.2	TTGTTCCTT	-281	-273	+	7.685	87.2	TTGTTCCTT
**NFATC2**	-281	-275	+	6.724	82.9	TGTTCCT	-280	-274	+	6.724	82.9	TGTTCCT
**Sox17**	-278	-270	+	5.776	81	TCCTTTGTT	-277	-269	+	5.776	81	TCCTTTGTT
**Sox2**	-277	-263	+	10.246	81.6	CCTTTGTTAATCACT	-276	-262	+	10.246	81.6	CCTTTGTTAATCACT
**HNF1A**	-273	-260	+	9.304	80.2	TGTTAATCACTGAC	-272	-259	+	9.304	80.2	TGTTAATCACTGAC
**AP1**	-271	-265	+	7.573	88.7	TTAATCA	-270	-264	+	7.573	88.7	TTAATCA
**Gfi**	-271	-262	+	9.241	90	TTAATCACTG	-270	-261	+	9.241	90	TTAATCACTG
**Nkx2-5**	-271	-265	+	5.339	84	TTAATCA	-270	-264	+	5.339	84	TTAATCA
**Pdx1**	-271	-266	+	5.014	81.3	TTAATC	-270	-265	+	5.014	81.3	TTAATC
**Nobox**	-270	-263	+	5.881	81.9	TAATCACT	-269	-262	+	5.881	81.9	TAATCACT
**CREB1**	-263	-256	+	6.22	80.4	TGACTTCT	-262	-255	+	6.22	80.4	TGACTTCT
**HIF1A::ARNT**	-247	-240	+	5.827	84	GGAGGTGC	-246	-239	+	5.827	84	GGAGGTGC
**HOXA5**	-229	-222	+	5.112	83.3	TGCTAATG	-228	-221	+	5.112	83.3	TGCTAATG
**Pdx1**	-227	-222	+	8.73	96.5	CTAATG	-226	-221	+	8.73	96.5	CTAATG
**Gata1**	-213	-203	+	4.964	82.4	TTGGATAAGGA	-212	-202	+	4.964	82.4	TTGGATAAGGA
**AP1**	-202	-196	+	7.794	89.5	TGACGCA	-201	-195	+	7.794	89.5	TGACGCA
**NR3C1**	-200	-183	+	8.953	80.9	ACGCACAGATTGTCCTAA	-199	-182	+	8.953	80.9	ACGCACAGATTGTCCTAA
**HOXA5**	-197	-190	+	5.427	84.4	CACAGATT	-196	-189	+	5.427	84.4	CACAGATT
**Sox17**	-195	-187	+	8.241	89	CAGATTGTC	-194	-186	+	8.241	89	CAGATTGTC
**SRF**	-189	-178	+	11.428	84	GTCCTAATAAGG	-188	-177	+	11.428	84	GTCCTAATAAGG
**Gata1**	-187	-177	+	3.954	80.6	CCTAATAAGGA	-186	-176	+	3.954	80.6	CCTAATAAGGA
**Pdx1**	-186	-181	+	6.478	87.3	CTAATA	-185	-180	+	6.478	87.3	CTAATA
**Nobox**	-185	-178	+	6.312	83.3	TAATAAGG	-184	-177	+	6.312	83.3	TAATAAGG
**Gata1**	-171	-161	+	4.475	81.5	ATTGAGAAAGG	-170	-160	+	4.906	82.3	ATTGAGAAAGA
**SPIB**	-169	-163	+	5.592	83.3	TGAGAAA	-168	-162	+	5.592	83.3	TGAGAAA
**Gata1**	-151	-141	+	6.204	84.7	TCTGAGAAGAT	-151	-141	+	7.096	86.3	TCTGAGAAGAG
**Nkx2-5**	-137	-131	+	4.492	80.6	ACAAGTC	-137	-131	+	4.492	80.6	ACAAGTC
**Gfi**	-136	-127	+	6.451	81.6	CAAGTCAGTG	-136	-127	+	6.318	81.2	CAAGTCAGAG
**Gata1**	-130	-120	+	3.927	80.5	AGTGAGAGGGC	-130	-120	+	5.157	82.8	AGAGAGAGGGC
**Klf4**	-124	-115	+	6.983	85.5	AGGGCGGGCA	-124	-115	+	6.983	85.5	AGGGCGGGCA
**Myb**	-118	-111	+	7.335	87.2	GGCAGTTT	-118	-111	+	7.335	87.2	GGCAGTTT
**HLF**	-96	-85	+	7.924	83.2	GATGACACAAGC	-96	-85	+	7.924	83.2	GATGACACAAGC
**AP1**	-94	-88	+	8.773	93.1	TGACACA	-94	-88	+	8.773	93.1	TGACACA
**CEBPA**	-94	-86	+	6.201	81.9	TGACACAAG	-94	-86	+	6.201	81.9	TGACACAAG
**CREB1**	-94	-87	+	6.329	80.8	TGACACAA	-94	-87	+	6.329	80.8	TGACACAA
**Nr2e3**	-89	-83	+	7.33	84.5	CAAGCAT	-89	-83	+	7.33	84.5	CAAGCAT
**Nkx2-5**	-84	-78	+	5.972	86.5	ATAAGTC	-84	-78	+	5.972	86.5	ATAAGTC
**ELF5**	-78	-70	+	10.352	96	CATTTCCTT	-78	-70	+	10.352	96	CATTTCCTT
**NFATC2**	-77	-71	+	7.175	84.6	ATTTCCT	-77	-71	+	7.175	84.6	ATTTCCT
**Pdx1**	-72	-67	+	5.029	81.3	CTTATT	-72	-67	+	5.029	81.3	CTTATT
**Nobox**	-71	-64	+	5.629	81.1	TTATTAAT	-71	-64	+	5.629	81.1	TTATTAAT
**Prrx2**	-70	-66	+	4.918	82.6	TATTA	-70	-66	+	4.918	82.6	TATTA
**ARID3A**	-69	-64	+	8.299	94	ATTAAT	-69	-64	+	8.299	94	ATTAAT
**Nkx2-5**	-68	-62	+	7.328	92	TTAATCG	-68	-62	+	9.317	100	TTAATTG
**Pdx1**	-68	-63	+	5.014	81.3	TTAATC	-68	-63	+	8.106	94	TTAATT
**Nobox**	-67	-60	+	6.919	85.3	TAATCGGT	-67	-60	+	11.363	100	TAATTGGT
**Tcfcp2l1**	-64	-51	+	11.265	89.6	TCGGTTCAAACCAG	-64	-51	+	8.52	85.6	TTGGTTCAAACCAG
**Tcfcp2l1**	-54	-41	+	9.483	87	CCAGTCCTTACAGG	-54	-41	+	10.401	88.3	CCAGTTCTTACAGG
**STAT1**	-50	-36	+	9.154	82.9	TCCTTACAGGAACTG	-50	-36	+	8.978	82.7	TTCTTACAGGAACTA
**Stat3**	-47	-38	+	9.082	85.5	TTACAGGAAC	-47	-38	+	9.082	85.5	TTACAGGAAC
**SPIB**	-45	-39	+	9.504	96.7	ACAGGAA	-45	-39	+	9.504	96.7	ACAGGAA
**FEV**	-44	-37	+	8.915	89	CAGGAACT	-44	-37	+	8.915	89	CAGGAACT
**GABPA**	-44	-34	+	7.913	84.5	CAGGAACTGGT	-44	-34	+	5.997	81.2	CAGGAACTAGT
**Myf**	-44	-33	+	12.077	91	CAGGAACTGGTG	-44	-33	+	7.989	81.6	CAGGAACTAGTG
**SPI1**	-43	-37	+	8.637	90.3	AGGAACT	-43	-37	+	8.637	90.3	AGGAACT
**MZF1_1-4**	-34	-29	+	5.838	85.2	TGGTGA	-34	-29	+	5.838	85.2	TGGTGA
**Gata1**	-33	-23	+	8.662	89.2	GGTGATAAATG	-33	-23	+	8.662	89.2	GGTGATAAATG
**HOXA5**	-30	-23	+	4.334	80.6	GATAAATG	-30	-23	+	4.334	80.6	GATAAATG
**ARID3A**	-29	-24	+	5.307	82.3	ATAAAT	-29	-24	+	5.307	82.3	ATAAAT
**Nkx2-5**	-29	-23	+	5.577	84.9	ATAAATG	-29	-23	+	5.577	84.9	ATAAATG
